# Safety and Feasibility of Steerable Radiofrequency Ablation in Combination with Cementoplasty for the Treatment of Large Extraspinal Bone Metastases

**DOI:** 10.3390/curroncol29080465

**Published:** 2022-08-20

**Authors:** Claudio Pusceddu, Davide De Francesco, Nicola Ballicu, Domiziana Santucci, Salvatore Marsico, Massimo Venturini, Davide Fior, Lorenzo Paolo Moramarco, Eliodoro Faiella

**Affiliations:** 1 Regional Referral Center for Oncologic Disease, Department of Oncological and Interventional Radiology, Businco Hospital, A.O. Brotzu, 09100 Cagliari, Italy; 2 Nima Aghaeepour Laboratory, Department of Radiology, Stanford University, Stanford, CA 94305, USA; 3 Department of Radiology, Sant’Anna Hospital, Via Ravona-22042, 88100 San Fermo della Battaglia, Italy; 4 Unit of Computer Systems and Bioinformatics, Department of Engineering, Campus Bio-Medico University, Via Alvaro del Portillo, 00128 Rome, Italy; 5 Department of Radiology, Hospital del Mar, 08003 Barcelona, Spain; 6 Department of Diagnostic and Interventional Radiology, University of Insubria, Ospedale di Circolo e Fondazione Macchi, 21100 Varese, Italy

**Keywords:** radiofrequency ablation (RFA), cementoplasty, bone metastases, steerable device

## Abstract

Background: Radiofrequency ablation (RFA) and cementoplasty, individually and in concert, has been adopted as palliative interventional strategies to reduce pain caused by bone metastases and prevent skeletal related events. We aim to evaluate the feasibility and safety of a steerable RFA device with an articulating bipolar extensible electrode for the treatment of extraspinal bone metastases. Methods: All data were retrospectively reviewed. All the ablation procedures were performed using a steerable RFA device (STAR, Merit Medical Systems, Inc., South Jordan, UT, USA). The pain was assessed with a VAS score before treatment and at 1-week and 3-, 6-, and 12-month follow-up. The Functional Mobility Scale (FMS) was recorded preoperatively and 1 month after the treatment through a four-point scale (4, bedridden; 3, use of wheelchair; 2, limited painful ambulation; 1, normal ambulation). Technical success was defined as successful intraoperative ablation and cementoplasty without major complications. Results: A statistically significant reduction of the median VAS score before treatment and 1 week after RFA and cementoplasty was observed (*p* < 0.001). A total of 6/7 patients who used a wheelchair reported normal ambulation 1 month after treatment. All patients with limited painful ambulation reported normal ambulation after the RFA and cementoplasty (*p* = 0.003). Technical success was achieved in all the combined procedures. Two cement leakages were reported. No local recurrences were observed after 1 year. Conclusions: The combined treatment of RFA with a steerable device and cementoplasty is a safe, feasible, and promising clinical option for the management of painful bone metastases, challenging for morphology and location, resulting in an improvement of the quality of life of patients.

## 1. Introduction

Bone is one of the most common metastatic sites for breast (70%), prostate (85%), and lung and kidney (40%) tumors, as well as multiple myeloma (MM) (95%) [1]. 

Bone metastases are associated with skeletal-related events (SREs) and complications such as pathological fracture, hypercalcemia, and bone marrow failure/leukoerythroblastic anemia [2].

SREs include cancer-induced bone pain, loss of mobility and social functioning, reduced quality of life (QoL), increased health care expenditure, and worse survival [3].

Available analgesic treatments for bone pain metastases, such as opioids that target the central nervous system, come with severe side effects, as well as the risk of abuse and addiction. Therefore, alternative interventional treatment options such as the targeted radiofrequency ablation (RFA) of bone metastases has become a standard procedure for the management of those bone cancer patients.

Furthermore, the value of radiofrequency ablation and cementoplasty, individually and in concert, has been adopted as palliative interventional strategies in the evidence-based National Comprehensive Cancer Network (NCCN) Practice Guidelines for Adult Cancer Pain. Specifically, regarding the RFA, the NCCN Guidelines for Adult Cancer Pain states “Ablative strategies such as radiofrequency ablation may also be performed to reduce pain and prevent skeletal related events (SREs)”. 

The RFA of bone lesions has proven successful for pain management in patients who are not candidates for open surgery and radiation therapy, and who receive inadequate relief from pharmacologic therapy [4].

Different thermal ablation modalities are actually available for the treatment of bone metastases; current percutaneous thermal ablation techniques used include cryoablation (CRA) and radiofrequency ablation (RFA), both of which are highly effective and safe in selected patients [5]. In most cases, RFA is technically performed with the insertion of only one electrode needle, which is a crucial advantage when there is a challenging location to be reached.

The choice of a steerable bipolar radiofrequency probe can result an essential tool for difficult-to-reach and large extraspinal bone metastases, and, when articulating the distal segment of the ablation probe into multiple portions of the tumor, can achieve a complete ablation area through a single osseous access channel.

This is a technical aspect that impacts on the choice of a steerable needle with respect to a fixed one.

The objective of this study is to evaluate the feasibility and safety of a steerable radiofrequency ablation device with an articulating bipolar extensible electrode (STAR, Tumor Ablation System, Merit Medical) in patients with painful extraspinal bone metastases assessing pain relief, as well as local tumor control in patients treated with RFA combined with cementoplasty.

## 2. Materials and Methods

### 2.1. Patient Population and Study Design

All of the single bone metastases percutaneous image-guided RFAs were performed at the department of Oncological and Interventional Radiology, Businco Hospital, A.O. Brotzu from February 2017 to June 2020 and were retrospectively evaluated. A total of 17 patients (6 men and 11 women) with an overall mean age of 63 years and an age range of 48–81 years were included in the study. The most common type of primary tumor was breast cancer in 12 patients (71%), followed by renal cell carcinoma (18%) in 3 patients. Other tumors included nonsmall-cell lung carcinoma and sarcoma. Patient and lesion characteristics are detailed in Table 1.

All patients were referred to the Interventional Radiology (IR) department after a multidisciplinary meeting because of pain resistant to conventional treatments, including opioids and radiotherapy, and not being candidates for the surgery. 

All patients were previously examined in the IR preprocedural consultation to explain modality and complications of the intervention. During the visit, a preliminary CT scan was requested and evaluated in order to select the eligible lesions. Only patients with lytic bone lesions that were technically challenging, with a standard straight ablative probe due to the location and irregular morphology within the bone, were included in the study. Patients with coagulopathy and an extra-osseous component were excluded. Informed consent for the procedure was obtained from all subjects.

The IRB approval was waived for this study due to its retrospective nature. 

All patients underwent radiofrequency ablation and cementoplasty in combination. The clinical decision to perform adjunct cementoplasty was based on the evaluation of biomechanics, fracture risk, location, and the type and extent of the lesion.

### 2.2. Radiofrequency Ablation (RFA) and Cementoplasty Procedure

Using CT fluoroscopy guidance (SOMATOM Sensation, Siemens, AG, Forchheim, Germany), the bone lesion was identified, measured, and marked to establish and validate the desired needle pathway. The procedures were performed according to the following steps: 

1. Anesthesia: Conscious sedation with intravenous infusion of fentanyl citrate (0.1 mg/2 mL diluted with saline solution) followed by local and periosteal anesthesia with lidocaine hydrochloride (2%). 

2. RFA: RFA was performed by using the STAR Tumor Ablation System (STAR, Merit Medical Systems, Inc., South Jordan, UT, USA) consisting of the steerable SpineSTAR^®^ ablation probe and the MetaSTAR generator. Under CT fluoroscopic guidance, a 10G introducer cannula, through which a steerable osteotome (PowerCURVE^®^ Navigating Osteotome) was inserted, was placed through the skin into the bone and used to create preferential channels for the targeted ablation. The RFA probe (SpineSTAR^®^ Ablation Instrument) was then advanced coaxially through the working cannula into the tumor to be treated for a targeted radiofrequency ablation. The steerable RFA probe has two thermocouples (the distal at 10 mm and the proximal at 15 mm from the ablation core) which actively monitor the ablation zone size by measuring the temperatures along the periphery of the ablation zone during the procedure. 

3. Cementoplasty: Through the same working cannula, high-viscosity bone cement with a long working time was slowly delivered (STABILIT, Merit Medical Systems, Inc.) for the optimal filling of the bone lesion. 

### 2.3. Follow-Up

Each patient was asked to quantify his or her pain with the VAS score before treatment. Subsequently, the VAS score was evaluated at 1-week and 3-, 6-, and 12-month follow-up outpatient office visits. 

The Functional Mobility Scale (FMS) was recorded preoperatively and 1 month after the treatment to assess the effect of treatment on level of mobility and ability to walk. A 4-point FMS classification was used: 4, bedridden; 3, use of wheelchair; 2, limited painful ambulation; 1, normal ambulation.

Technical success was defined as a successful intraoperative ablation and cementoplasty without any major complications. Major and minor complications were evaluated based on the CIRSE classification system [6].

Local tumor control was assessed with a CE-CT scan and or CE-MRI performed 12 months after the treatment and defined as the absence of viable tissue enhancing at imaging within the entire tumor treated.

### 2.4. Statistical Analysis

Median and interquartile ranges (IQR) for the VAS score at each visit (before RFA and 1 week, 1 month, 3 months, 6 months and 12 months after RFA) were calculated. The significance of the change from one visit to the following visit was assessed using the Wilcoxon test for paired samples. The distribution of the FMS classification before and after the RFA was evaluated using the McNemar test. 

Analyses were performed using the statistical software R v4.0.2 and *p*-values < 0.05.

## 3. Results

**Figure 1:**
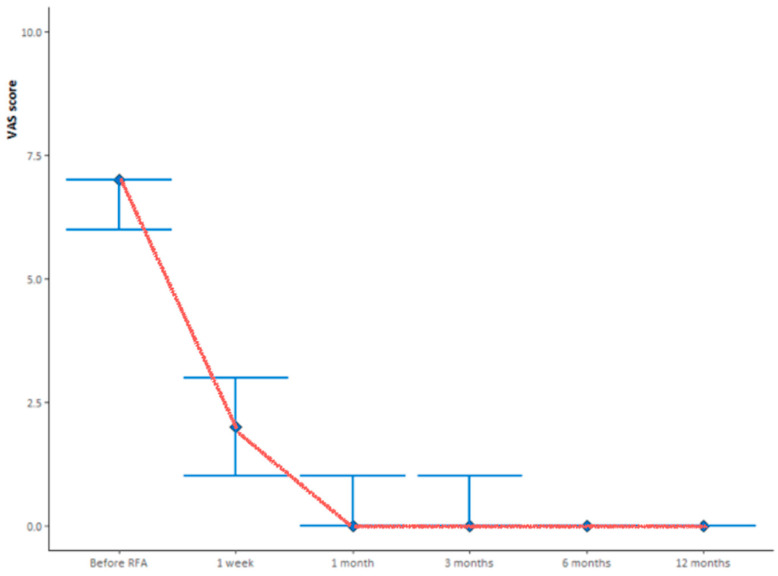
Median VAS score follow-up evaluated before and prospectively after 1 week, and 1, 3, 6 and 12 months from the treatment. The red line indicates patients’ changes in pain.

The change over time of the median VAS score in the study population is shown in Figure 1. While the median (IQR) VAS score before the RFA was 7 (6, 7), 1 week after the RFA it dropped to 2 (1, 3). The five-point reduction was statistically significant (*p* < 0.001). At the following visits, the median (IQR) VAS score was 0 (0,1) at 1 month after the RFA (*p* = 0.009 compared to 1 week after RFA), 0 (0, 1) at 3 months after the RFA, 0 (0, 0) at 6 months after the RFA, and 0 (0, 0) at 12 months after the RFA. The change from 1 month to 3 months after the RFA (*p* = 0.09), from 3 months to 6 months after the RFA (*p* = 0.86), and from 6 months to 12 months after the RFA (*p* = 0.99) was not significant.

**Figure 2:**
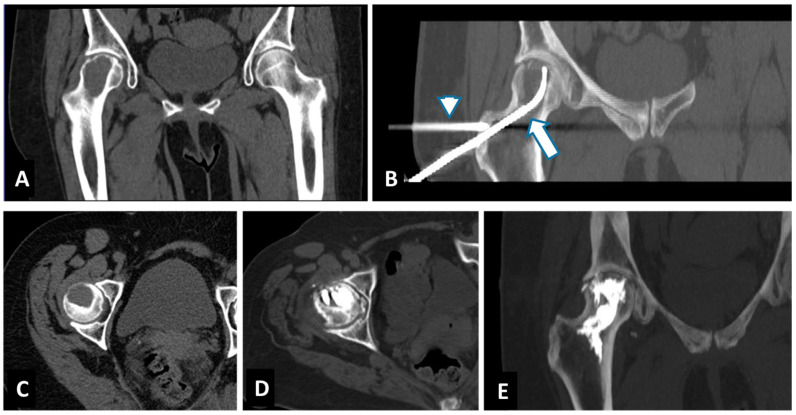
Successful treatment with tRFA and osteoplasty of painful breast cancer metastases of the right femur in a 61-year-old woman. Axial CT and Coronal-CT reconstruction showed a 52 mm lesion located in the head and neck of the right femur (**A**,**C**). Coronal-CT reconstruction showed the placements of the steerable bipolar radiofrequency device (tSTAR) (arrow). A second cannula is placed through the greater trochanter to extend osteoplasty (arrowhead) (**B**). Axial and Coronal-CT reconstruction showed the result after osteoplasty with PMMA (**D**,**E**).

Before the RFA, 3 (17.6%), patients reported normal ambulation on the FMS, seven (41.2%) reported limited painful ambulation, and seven (41.2%) reported the use of a wheelchair (Figure 2). Six of the seven patients who reported the use of a wheelchair before the RFA reported normal ambulation 1 month after the RFA, with the remaining patients reporting limited painful ambulation. All seven patients who reported limited painful ambulation before the RFA improved mobility and reported normal ambulation after RFA (Table 2). The observed improvement in mobility, which was statistically significant (*p* = 0.003).

Technical success was achieved in all the combined procedures. Two cement leakages for the sacrum and femur metastases and no other major complications were reported after the treatment.

## 4. Discussion

A steerable radiofrequency ablation device with an articulating bipolar extensible electrode in combination with cementoplasty is a feasible and safe option for the treatment that is challenging for the location and morphology lytic metastatic bone lesions. The role of the ablative technique for the minimally invasive treatment of bone metastases are nowadays scientifically recognized as an important clinical option for tumor patients. Recent studies demonstrated the role of the bone marrow microenvironment (e.g., osteoclasts, osteoblasts, macrophages, mast cells, mesenchymal stem cells, and fibroblasts) in the development of cancer-induced bone pain [7]. Proliferating tumor cells in the bone microenvironment produce a range of cytokines and growth factors that increase the osteoblast production of the receptor activator of nuclear factor kappa B ligand (RANKL). This leads to activation of osteoclasts and the disturbance of the normal coupling of bone formation and bone resorption. Bone-derived growth factors are released during bone resorption and may stimulate the proliferation of the tumor cell population, and thus create a self-sustaining vicious cycle between cancer cells and the bone microenvironment [8].

The clinical value of the percutaneous RFA when performed in stage IV cancer patients with painful bone metastases has far reaching clinical utility as a component of a comprehensive treatment algorithm in bone metastatic disease. As a nonionizing ablative-therapy, RFA has been documented to reduce tumor burden and provide significant, rapid and durable pain relief within hours/days, demonstrating that patients with painful bone metastases benefit from an RFA treatment option when [9,10]:

- radiation is no longer an option because the maximum tolerable dose has been reached;
- pain prevents lying in the prone position for radiation therapy planning and course of treatment;
- RFA can be provided as an adjunct to conventional fractionated external beam radiotherapy [11], which requires multiple fractions and 4-6 weeks to achieve a palliative response [12];
- tumor histology is a radiation-resistant tumor and stereotactic body radiation therapy (SBRT) is not available.

The European Society for Medical Oncology (ESMO) Clinical Practice Guidelines refers to several ablative therapeutic options available for patient suffering from bone metastases, such as cryoablation, radiofrequency, and microwave ablation [13].

A multidisciplinary team discussion is required to assess the correct clinical and therapeutic pathway for patients with bone metastases and to select the most appropriate thermal ablative approach [6].

The aim of minimally-invasive ablation treatment is addressing the biological pain due to the stretching and irritation of the periosteum secondary to tumor growth and due to osteoclast-mediated bone resorption with the release of neurostimulating cytokines. The purpose of cementoplasty is to treat the mechanical pain for the instability from pathologic microfractures [14,15].

Minimally invasive RFA treatment in combination with cementoplasty offers a fast and sustained long-term improvement in pain and quality of life, as reported by previous scientific manuscripts and confirmed in this study, which have demonstrated the safety and the rapid pain relief after the RFA for bone tumors [16,17]. 

The percutaneous approach to a bone lesion can sometimes be particularly complex based on the lesion anatomical site and morphology. Furthermore, to obtain a large enough ablation area, such as to include the pathological tissue, it is often necessary to insert more than one electrode needle. In most cases, a percutaneous approach using one or more working cannulas is technically feasible, but it can represent a procedural bet when the lesion is localized in complex locations, when it is necessary to respect the load or force lines of the bone segment, or to avoid damaging adjacent muscular or nerve structures. In such situations, it is particularly advantageous to reach the lesion through a single safe access and to have a steerable ablation device instead of a fixed tip. In load sites, such as the bony structures of the pelvis, it becomes crucial to use a direct access to the lesion that can preserve and follow the normal load lines (such as the neck of the femur, rather than the ischium or ileus branches/pubic) (Figure 2).

**Figure 3:**
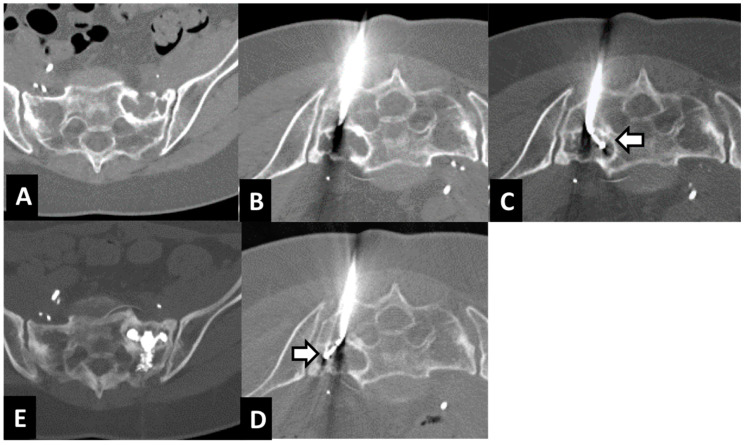
Treatment with tRFA and osteoplasty of a sacral metastasis from breast cancer in a 62-year-old woman. Axial CT scan showed a 37 mm lytic metastasis of the left sacral ala (**A**). The metastasis was reached with a 10-gauge Stabilit cannula (**B**). Axial CT showed two different placements of the steerable radiofrequency bipolar device (tSTAR) covering the entire lesion (arrows) (**C**,**D**). Axial CT scan showed the metastasis filled by PMMA (**E**).

The choice of a steerable bipolar radiofrequency probe for difficult-to-reach extraspinal bone metastases was based on the advantages of articulating the distal segment of the ablation probe into multiple portions of the tumor from a single osseous access channel [18] (Figure 3). 

As reported in the literature, some extra-spinal bone metastases are technically challenging to approach [17]. The navigational tip of the probe, instead of a fixed one, can be directed in different placements through the same access introducer for accessing lesions in challenging locations, as well as achieving larger overlapping ablation zones [19].

The use of a steerable RFA probe in combination with a high-viscosity cement has been demonstrated as a feasible and safe procedure in the difficult-to-reach extra-spinal bone metastases. To our knowledge, this is the first study which investigates the RFA/cementoplasty approach through this specific needle.

**Figure 4:**
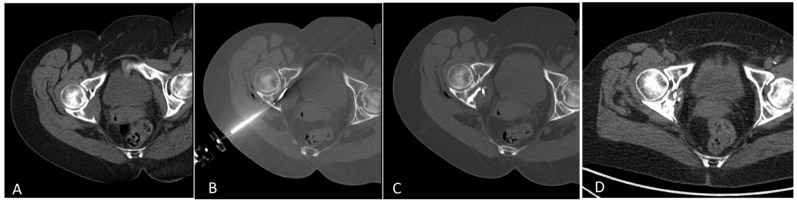
Treatment with tRFA and osteoplasty of an osteolytic metastasis of the posterior column of the right hip from breast cancer in a 65-year-old woman (**A**). Axial CT showed the placement of the steerable radiofrequency bipolar device (tSTAR) (**B**). Axial CT scan showed the metastases filled by PMMA (**C**). Axial CT after 12 months showed a partial recalcification of the posterior column of the hip and disappearance of the tumor mass (**D**).

The pain relief outcome of this study is aligned with the results of the recent literature and with the increasing evidence in oncology that survival is linked to symptom reporting and control, and that pain management contributes to broad quality-of-life improvement [12]. In particular, we observed an immediate pain relief after the procedure in all patients, with a VAS dropping to 0 after 1 week in 53% of patients, and which was lower in 100% of patients. The recovery of walking (normal ambulation) was successfully achieved at 1-month follow-up after the interventional treatment in 94% of patients with a great improvement on the wheelchair patient group (Figure 4).

Our results are similar to those reported in the literature which evaluated the combined RFA/cementoplasty approach but with a traditional needle. 

In the paper of Fares et al., which evaluated extraspinal metastases, the VAS score decreased significantly after 1 day, and at 1, 4, and 12 weeks (preoperative: 72.3; day 1: 31.5; week 1: 32.5; week 4: 36.7; week 12: 45.6) [20]. 

Nakatsuka and colleagues evaluated 17 patients with 23 mainly spine metastatic lesions and reported pain relief within one week in all patients and one failure in one patient with an osteoblastic lesion [21].

Other retrospective analyses showed a pain reduction in all 25 patients (with minor complications in 20%), in 92.1% on 38 patients, and in 100% on all patients with acetabular metastases, respectively [17,22,23].

This study has several limitations, due to the retrospective nature and to the small number of patients; however, we demonstrated an alternatively valid approach for those metastatic bone lesions that are difficult-to-treat with a traditional fixed-tip needle. Other studies should be performed including patients with different kind of metastases.

## 5. Conclusions

In conclusion, the combined treatment of RFA with a steerable device and cementoplasty is a safe, feasible, and promising clinical option for the management of painful bone metastases that are challenging for their morphology and location, resulting in an improvement of the quality of life of patients.

## Figures and Tables

**Table 1:** Patients and tumor characteristics.

Age—Gender	Primary Tumor	Site of Lesion	Size of Lesion mm	Mobility Recovery	VAS before RFA	VAS after: 1 w, 1 m, 3 m, 6 m, 12 m	Cementoplasty	Complication	Imaging after 1 y Follow-Up
70—W	Breast	left acetabulum	35 × 30 × 25 mm	2 1	7	3 0 0 0 0	7 cc	leakage cement	CT
62—W	Breast	left ilium	12 × 8 × 19 mm	2 1	6	3 0 0 0 0	4 cc	none	MRI
79—M	Renal cell carcinoma	left ilium	32 × 35 × 30 mm	3 1	8	2 0 0 2 1	4 cc	none	CT
61—W	Breast	left femur	25 × 25 × 30 mm	3 1	7	1 0 0 1 N/A	4 cc	none	CT
67—W	Breast	right ilium	30 × 22 × 37 mm	3 1	6	0 0 0 0 0	8 cc	none	CT
59—W	Breast	left acetabulum + quadrilateral lamina	34 × 34 × 18 mm	3 2	8	4 4 2 0 0	5 cc	none	CT
81—W	Breast	right acetabulum	10 × 15 × 20 mm	3 1	7	1 0 1 2 N/A	5 cc	none	CT-MRI
57—M	Non-small-cell lung carcinoma	right ilium	15 × 14 × 18 mm	1 1	6	0 1 1 0 0	4 cc	none	CT
69—M	Breast cancer	right humerum	25 × 25 × 30 mm	3 1	8	2 2 0 0 0	6 cc	none	CT
69—M	Renal cell carcinoma	sacrum	30 × 32 × 28 mm	2 1	7	3 1 0 0 0	4 cc	none	CT
55—M	Renal cell carcinoma	left acetabulum	18 × 15 × 25 mm	2 1	6	1 1 0 0 0	4 cc	none	CT
61—W	Breast	right femur	35 × 25 × 52 mm	3 1	9	3 0 0 0 0	10 cc	leakage cement	CT-MRI
48—W	Breast	left ilium	30 × 31 × 34 mm	2 1	6	2 1 1 0 1	7 cc	none	CT
66—M	Sarcoma	sacrum	28 × 20 × 33 mm	1 1	5	0 0 0 0 N/A	6 cc	leakage cement	CT
63—W	Breast	sacrum	30 × 25 × 35 mm	1 1	7	1 0 0 0 0	6 cc	none	CT
62—W	Breast	right acetabulum	20 × 20 × 32 mm	2 1	5	2 1 1 0 0	7 cc	none	CT
62—W	Breast	sacrum	25 × 22 × 37 mm	2 1	7	2 2 0 0 0	7 cc	none	CT

**Table 2:** Between walking performance rated on the Functional Mobility Scale (FMS) and measures of walking capacity after the treatment.

		1 Month after RFA			
		Normal Ambulation	Limited Painful Ambulation	Use of Wheelchair	Bedridden
**Before** **RFA**	**Normal ambulation**	3	0	0	0
	**Limited painful ambulation**	7	0	0	0
	**Use of wheelchair**	6	1	0	0
	**Bedridden**	0	0	0	0

## Data Availability

The data presented in this study are openly available in references.
